# A 3D environment influences osteocyte function

**DOI:** 10.1186/1753-6561-9-S1-A7

**Published:** 2015-01-14

**Authors:** Yuan-Hsun Chang, RT Brady, O Brennan, FJ O’Brien

**Affiliations:** 1 Tissue Engineering Research Group, Royal College of Surgeons in Ireland, Dublin 2, Ireland; 2 Trinity Centre for Bioengineering, School of Engineering, Trinity College Dublin, Ireland; 3 Advanced Materials and BioEngineering Research Centre (AMBER), Trinity College Dublin, Ireland

## Background

Osteocytes are critical in bone maintenance, adaptation and have important endocrine functions including mineral homeostasis through osteocyte-specific factors such as fibroblast growth factor 23 (fgf-23), a regulator of serum phosphate. MLO-Y4 cells are an osteocyte—like cell line that expresses negligible levels of fgf23. To date, no study has yet investigated the effect of a 3-dimensional culture system upon MLO-Y4 cells. The overall study objective was therefore to examine the effects of 3D culture upon MLO-Y4 expression of fgf23 using a collagen-glycosaminoglycan (GAG) 3D scaffold. In addition the effect of mechanical cues, scaffold stiffness and fluid flow shear stress, in directing cell behaviour was also studied.

## Methods

MLO-Y4 cells were cultured upon 3D collagen-GAG scaffolds. Mechanical stimuli effects were applied by varying the scaffold substrate stiffness and using a perfusion bioreactor to apply fluid flow shear stress. Scaffolds were separated into static culture groups or flow group. Real-time PCR was used to determine Cox2 and Fgf-23 expression. The mechanosensitive gene Cox2 was used to validate the applied mechanical cues experienced by the osteocytes.

## Results

Results indicate that MLO-Y4 cells were found to express fgf23 when cultured on a 3D scaffold compared to a 2D control with gene expression significantly raised with increasing scaffold stiffness. The addition of fluid flow resulted in higher gene expression compared to statically cultured controls. Results were validated by increased expression of Cox-2 with increasing scaffold stiffness and fluid flow.

## Conclusions

This is the first study to show 3D collagen-GAG scaffolds, can direct osteocyte function. Increasing substrate stiffness augmented expression of the aforementioned genes. Flow stimulation further enhanced gene expression. In conclusion, we have demonstrated that 3D culture can influence osteocyte biology, promoting the expression of fgf23. We have also shown that both substrate stiffness and fluid flow can significantly influence osteocyte gene expression, demonstrating that fgf23 is a mechanically regulated protein. This data further highlights the importance of mechanical cues in directing cell behaviour, the finding that fgf23 is mechanically regulated has important implications regarding a mechanically regulated endocrine axis.

